# Microbiota, type 2 diabetes and non-alcoholic fatty liver disease: protocol of an observational study

**DOI:** 10.1186/s12967-019-02130-z

**Published:** 2019-12-04

**Authors:** Benedetta M. Motta, Christoph Grander, Martin Gögele, Luisa Foco, Vladimir Vukovic, Roberto Melotti, Christian Fuchsberger, Alessandro De Grandi, Chiara Cantaloni, Anne Picard, Deborah Mascalzoni, Alessandra Rossini, Cristian Pattaro, Herbert Tilg, Peter P. Pramstaller

**Affiliations:** 1 Institute for Biomedicine (affiliated to the University of Lübeck), Eurac Research, 39100 Bolzano, Italy; 2 Department of Internal Medicine I, Gastroenterology, Hepatology, Endocrinology & Metabolism, Medical University Innsbruck, Innsbruck, Austria; 3 Center for Research Ethics and Bioethics, Department of Public Health and Caring Science, Uppsala University, Uppsala, Sweden

**Keywords:** Microbiota, Type 2 diabetes, NAFLD, Cooperative Health Research In South Tyrol

## Abstract

**Background:**

Non-alcoholic fatty liver disease (NAFLD) is characterized by triglyceride accumulation in the hepatocytes in the absence of alcohol overconsumption, commonly associated with insulin resistance and obesity. Both NAFLD and type 2 diabetes (T2D) are characterized by an altered microbiota composition, however the role of the microbiota in NAFLD and T2D is not well understood. To assess the relationship between alteration in the microbiota and NAFLD while dissecting the role of T2D, we established a nested study on T2D and non-T2D individuals within the Cooperative Health Research In South Tyrol (CHRIS) study, called the CHRIS-NAFLD study. Here, we present the study protocol along with baseline and follow-up characteristics of study participants.

**Methods:**

Among the first 4979 CHRIS study participants, 227 individuals with T2D were identified and recalled, along with 227 age- and sex-matched non-T2D individuals. Participants underwent ultrasound and transient elastography examination to evaluate the presence of hepatic steatosis and liver stiffness. Additionally, sampling of saliva and faeces, biochemical measurements and clinical interviews were carried out.

**Results:**

We recruited 173 T2D and 183 non-T2D participants (78% overall response rate). Hepatic steatosis was more common in T2D (63.7%) than non-T2D (36.3%) participants. T2D participants also had higher levels of liver stiffness (median 4.8 kPa, interquartile range (IQR) 3.7, 5.9) than non-T2D participants (median 3.9 kPa, IQR 3.3, 5.1). The non-invasive scoring systems like the NAFLD fibrosis score (NFS) suggests an increased liver fibrosis in T2D (mean − 0.55, standard deviation, SD, 1.30) than non-T2D participants (mean − 1.30, SD, 1.17).

**Discussion:**

Given the comprehensive biochemical and clinical characterization of study participants, once the bioinformatics classification of the microbiota will be completed, the CHRIS-NAFLD study will become a useful resource to further our understanding of the relationship between microbiota, T2D and NAFLD.

## Background

Non-alcoholic fatty liver disease (NAFLD) encompasses a spectrum of disorders characterized by hepatic triglyceride accumulation (hepatic steatosis) in the absence of alcohol overconsumption [1]. Twenty-to-thirty % of the NAFLD patients progress to non-alcoholic steatohepatitis (NASH), implying liver inflammation and association with liver-related diseases such as fibrosis, cirrhosis, and hepatocellular carcinoma [2]. NAFLD has an estimated prevalence of about 25% in the general population [3] and is expected to become the leading cause of liver transplantation over the next 20 years, with expanding costs for healthcare systems [4]. In the presence of type 2 diabetes (T2D) and metabolic syndrome (MetS), NAFLD prevalence can rise up to 70% [5–7]. T2D may increase the risk of developing NAFLD and NASH, but also NAFLD itself may be a risk factor for T2D onset [8, 9]. The interplay between NAFLD, T2D, and MetS is complex, with NAFLD and MetS sharing clinical manifestations such as obesity, insulin resistance, T2D, dyslipidemia, and hypertension [10].

Triggering factors, such as the translocation of bacterial components and their products from the gut into the systemic circulation following alterations of the intestinal integrity, have been identified as an important mechanism of NAFLD onset [11, 12]. Animal studies suggest that bacterial components may also have a crucial role in NAFLD and NASH onset in humans [13].

The gut microbiota represents the community of microorganisms inhabiting the digestive tract, with vital functions in relation to vitamin biosynthesis, bile acid degradation, maintenance of the intestinal mucosal barrier integrity, and complex carbohydrate digestion. Microbiota composition can be altered by medications, environmental factors, and diet [14, 15]. Individuals with T2D [16–18] or MetS [19] may suffer a disrupted intestinal microbial composition, which then promotes an imbalance between protective and harmful effects of the microbiota on the host. Increasing evidence suggests an important role for the intestinal microbiota in the pathogenesis of T2D, regulating metabolic pathways and glucose hemostasis [20]. For example, obesity is associated with a larger number of bacterial strains that ferment food components and increase the potential of the host to harvest energy [21]. Obese and MetS-affected individuals may have an altered ratio of Firmicutes-to-Bacteroidetes specific phyla [22, 23]. Members of both phyla are involved in the production of short chain fatty acids (SCFAs) from dietary compounds not completely digested in the small intestine [24]. Evidence suggests a role of SCFAs as key mediators of the cross talk between brain and gut in the pathogenesis of obesity [25]. It has been shown that the total amount of SCFA produced is higher in obese subjects, suggesting that SCFA metabolism might play a considerable role in obesity [26]. Recently, using bidirectional Mendelian randomization, the causal relationship between the gut microbiome and metabolic traits has been explored, providing evidence of a causal effect of the gut microbiome on metabolic traits [27]. Several studies have shown alterations of the microbiota composition in NAFLD [28–30] and NASH [31] patients. Increased relative abundance of Bacteroides and *Ruminococcus* in the intestine has been associated with more severe histology in NAFLD patients [32]. In contrast, NAFLD patients seem to have lower relative abundance of the *Prevotella* strain [32, 33]. Recently, 37 bacterial strains from the gut were identified that allowed discrimination between mild and severe hepatic fibrosis in biopsy-proven NAFLD patients [34].

Another important microbial habitat is the oral cavity [35]. In addition to contributing to oral diseases [36, 37], the oral microbiota may represent a risk factor for systemic diseases such as T2D [38, 39]. NAFLD was associated with periodontitis, and some characteristics of periodontitis such as systemic inflammation and invasion of the commensal bacteria are involved in progression of liver fibrosis in NAFLD-affected individuals [40]. Experimental models showed an association between *Porphyromonas gingivalis* and risk of NAFLD and NASH [41, 42]. The importance of microbial invasion from the oral cavity into the lower intestine compartments in patients with cirrhotic liver disease was recently demonstrated by a study showing that > 50% of bacterial species found to be enriched in the intestine of cirrhotic patients were of buccal origin [43]. While the oral microbiota has gained much attention only recently, there remain many unanswered questions on the role of bacterial strains on specific pathologies of the liver.

To further improve the general understanding on the relationship between the microbiota, from gut and mouth, and NALFD, and to illuminate such relationships in the context of T2D, we carried out an observational study nested within the Cooperative Health Research in South Tyrol (CHRIS) study [44], called CHRIS-NAFLD. Here, we describe the study protocol, recruitment and measurement procedures, and offer a description of the epidemiological characteristics of study participants at the baseline and follow-up times.

## Methods

### Study design

The CHRIS-NAFLD study was setup in the context of the CHRIS study, a population-based study carried out in a rural Alpine context [44, 45]. CHRIS study participants were on overnight fasting, underwent blood and urine collection, anthropometric and blood pressure (BP) measurements, electrocardiographic analysis, and tremor assessment. Participants were also administered a series of interviewer- and self-administered questionnaires about their health status. The CHRIS-NAFLD study was designed after the recruitment of the first 4979 CHRIS study participants, which was carried out between 2011 and 2014 (baseline data) [44]. From the 4979 participants, we selected for the CHRIS-NAFLD study all 227 individuals affected by T2D and an equal number of non-T2D individuals, matched on age (± 2 years tolerance) and sex. T2D was defined according to standard guidelines [46], as a positive response to the question “Has a doctor ever diagnosed you with diabetes?” or either having fasting plasma glucose levels of ≥ 126 mg/dl or glycated hemoglobin (HbA1c) levels of ≥ 6.5%. Participants with other types of diabetes were excluded from the selection. Among participants selected as non-T2D diabetes (negative response to the doctor’s diagnosed diabetes question), participants were further excluded in case of HbA1c levels beyond 5.6%, to prevent inclusion of potentially pre-diabetic participants.

### Recruitment of study participants

Selected participants were informed about the objective and content of the CHRIS-NAFLD study by invitation letter, mailed between 2 and 3 weeks prior to enrollment. They were subsequently contacted by phone to arrange an appointment at the study center. The participants were requested not to eat anything from 8:00 pm of the night before the day of participation, and to abstain from drinking or smoking for at least 2 h prior to the visit. At the study center, participants underwent blood drawing, urine collection, saliva sampling, anthropometric and BP measurements, tremor assessment, and clinical examination by a medical doctor to assess the hepatic status. Body mass index (BMI), fat percentage, and visceral and subcutaneous fat were assessed using a body composition monitor (OMRON BF508). Waist and hip circumference were measured according to the WHO protocol [47].

### Questionnaires and interview

Questionnaires concerning the change of participants’ health status since the baseline participation and their life-style were administered by an interviewer, and the medication intake in the last 7 days was documented using an electronic optical scan of their medication box bar codes according to the Anatomical Therapeutic Chemical (ATC) classification system. A Food Frequency Questionnaire (FFQ) based on the Global Allergy and Asthma European Network of Excellence study [48] was mailed to their homes prior to participation, in order to limit the time spent at the study center. The FFQ also asked about the average frequency of the consumption of alcoholic drinks during the last 12 months (rarely or never, 1–3/month, 1/week, 2–4/week, 5–6/week, 1/day, 2+/day), specifically of beer (200 ml), red wine (125 ml), white wine (125 ml), rosé wine (125 ml), liqueurs (50 ml), and spirits (50 ml). At the study center, participants answered a computer-assisted interviewer-administered questionnaire on smoking habits, based on the European Community Respiratory Health Survey II [49] from which we derived pack-years as a measure of cumulative smoking. Based on their smoking habits, participants were classified as never smokers (never smoked or smoked for < 1 year in their lifetime), past-smokers (smoked for ≥ 1 year in their lifetime but stop smoking ≥ 1 year before the interview), and current-smokers (currently smoking at the time of the interview or stopped smoking < 1 month before the interview).

### Evaluation of the hepatic steatosis and fibrosis

To evaluate the presence of hepatic steatosis and fibrosis, participants underwent abdominal ultrasound examination (5-1 MHz Phased Array Transducer, iViz, SonoSite, USA) and transient elastography (Fibroscan^®^, Echosens, France) performed by a trained medical doctor (Christoph Grander).

To assess transient elastography (TE), participants were placed in a supine position with their right arm fully adducted and asked to hold their breath. At least ten independent resistance measurements were taken, starting always with an M + probe but using an XL + probe as a backup option when prompted by the automatic probe selection tool [50, 51]. TE values were defined as unreliable when the IQR to median ratio was > 30%. Fibrosis was then scaled into four stages, F0 to F4, based on the resistance levels of the liver [52]. TE values of > 6.5 kPa were considered as diagnosis of fibrosis (≥ F1) [53].

Additionally, abdominal ultrasound scanning was performed in every participant after overnight fasting. Presence and severity of steatosis was evaluated as documented by Ballestri et al. [54]. Steatosis was classified into three grades: normal or very slight increase in the echo pattern with normal visualization of vessels and diaphragm (grade 1); moderate increase in echogenicity with reduced visibility of portal veins and diaphragm (grade 2); or distinct increase in echo pattern with poor visibility of intrahepatic vessels and diaphragm (grade 3). Presence of gallstones, gallbladder size, and wall thickness were also assessed, as well as the visceral and subcutaneous fat thickness to gain insights into the participant’s metabolic risk profile [55].

The presence of NAFLD was defined as steatosis grade of ≥ 2, after the exclusion of other causes such as overt hepatitis due to virus infection, or hereditary liver disorders or other liver diseases [56]. Three participants were excluded from further analyses because of potential drug induced steatosis by methotrexate. Participants were not excluded based on alcohol consumption levels.

Advanced fibrosis was defined as LSM values ≥ 6.5 kPa

For a deeper characterization of steatosis and fibrosis, additional surrogate markers have been calculated as reported in Box 1.

Box 1. Evaluation of MetS and hepatic steatosis/fibrosis Metabolic syndrome (MetS) [57, 58]. Presence of 3 risk factors:

Must have:

Central obesity (WC ≥ 94 cm in males and ≥ 80 cm in females).

Plus any two of the following four factors:

- TG level: ≥ 150 mg/dl (1.7 mmol/l), or specific treatment for this lipid abnormality.
- HDL cholesterol: < 40 mg/dl (1.03 mmol/l) in males and < 50 mg/dl (1.29 mmol/l) in females, or specific treatment for this lipid abnormality.
- Systolic BP ≥ 130 or diastolic BP ≥ 85 mm Hg, or treatment of previously diagnosed hypertension.
- FPG ≥ 100 mg/dl (5.6 mmol/l), or previously diagnosed T2D.

*Visceral adiposity index (VAI)* [59]

Males: (WC [cm]/39.68 + 1.88 · BMI [kg/m²]) · TG [mmol/l]/1.03 ·1.31/HDL [mmol/l]

Females: (WC [cm] /36.58 + 1.89 · BMI [kg/m²]) · TG [mmol/l] /0.81 · 1.52/HDL [mmol/l]

*NAFLD liver fat score (LFS) *[60]

NAFLD-LFS: − 2.89 + 1.18 · MetS [yes = 1, no = 0] + 0.90 · T2D [yes = 1, no = 0] + 0.15 · insulin [mU/l] + 0.04 · AST [U/l] – 0.94 · AST [U/l] / ALT [U/l]

*Hepatic steatosis score (HSI) *[61]

HSI: 8 · ALT [IU/l]/AST [IU/l] + BMI [kg/m²] (+2 if T2D; +2 if female)

*Fatty liver index (FLI) *[62]

FLI = e^θ^/ (1 + e^θ^) · 100, where θ = 0.953 · ln(TG [mmol/l]) + 0.139 · BMI [kg/m²] + 0.718 · ln(GGT [U/l]) + 0.053 · WC [cm] – 15.745

*NAFLD fibrosis score (NFS)* [63]

NFS: −1.675 + 0.037 · age [years] + 0.094 · BMI [kg/m^2^] + 1.13 · IFG or DM [yes = 1, no = 0] + 0.99 · AST/ALT − 0.013 · PLT [×10^−9^/l] − 0.66 · albumin [g/dl]

*Fibrosis-4 (FIB-4)* [64]

Fib-4: (age [years] · AST [U/l]) / (PLT [10^9^/l] · ALT [U/l]^1/2^)

*Homeostatic model assessment-insulin resistance (HOMA-IR)* [65]

HOMA-IR: FPG [mg/dl] · insulin [mU/l]/405

### Biospecimen collection and biobanking

For the CHRIS-NAFLD study, blood (49 ml) and urine (30 ml) samples for laboratory analysis and biobanking were collected in the early morning, after an overnight fasting, following the same procedures previously described for the CHRIS study in terms of sample pre-analytical processing, transportation, and biobanking [44, 45]. In addition, two 1 ml aliquots of serum were stored at − 80 °C and sent in dry ice in a unique batch at the end of the recruitment to Synlab Italia Srl for insulin measurement. The CHRIS biobank was assigned a “Bioresource Research Impact Factor” code BRIF6107 [44, 66].

### Stool and saliva collection for the metagenomic analysis

Stool collection tubes were shipped to the participant’s home some days before the enrollment with instructions for the sample collection. Participants were asked to collect the samples in the same morning of their visit or, failing that, within 24 h of the visit. Participants brought their stool samples to the study center at room temperature. Once at the study center, samples were stored at − 20 °C. Samples were then transported frozen to the biobank, where they were finally stored at − 80 °C. Information about the exact time of defecation was collected and the Bristol stool scale (BSS) was assessed [67]. The BSS is used to classify stool consistence: it can be used as a surrogate marker for stool transit time [68] and is applied in both clinical and experimental fields [69].

Non-stimulated saliva samples were collected using the Omnigene oral collection device (OM-501, DNA Genotek, USA) at the study center. After collection, stabilized saliva samples were transported to the biobank, where they were stored at − 80 °C after splitting into 500 μl aliquots. Status of teeth and gums was assessed using items 3, 6, and 12 of the WHO’s Oral Health Questionnaire (OHQ) for Adults [70].

### Microbiome extraction protocol

DNA extraction from the faeces was performed using a Chemagic Magnetic Separation Module I Dispenser (Perkin Elmer, USA) according to the Chemagen protocol (Chemagic DNA Feces 1 k drying prefilling H12 VD160617.che) using a blood kit (CMG-763-1, Perkin Elmer) supplemented with a lysis buffer specifically for stools (CMG-852, Perkin Elmer). Briefly, under a sterile hood, up to 1 g of each sample was taken and immediately immersed in 8 ml lysis buffer. The weight was annotated and the tube mixed thoroughly on a vortex. After adding 50 μl protease mix (provided in the kit), the sample was incubated for 20 min at 70 °C followed by 5 min inactivation at 95 °C. The sample was centrifuged and the supernatant transferred to a new tube that was further processed on the robot.

For the DNA extraction protocol from saliva, based on chemical lysis and purification for downstream applications, we followed the manufacturer’s protocol (CMG-1037, Chemagic DNA Saliva Kit special, Perkin Elmer). DNA was quantified with QuantiFluor (E2670, Promega) on an Envision plate reader (Perkin Elmer) and quality was assayed on a NanoDrop spectrophotometer (ThermoFisher, USA) and by running on a 0.5% agarose gel.

Metagenomic sequencing will be based on the amplification of the V3–V4 hypervariable region of the 16S RNA gene with specific primers which can then be used to incorporate unique indexes into the fragments which will further allow unique indexing of up to 384 samples that can then be pooled together. The resulting multiplexed pool will be run on the MiSeq System using the V3 chemistry 600 cycle kit (16S Metagenomic Sequencing Library Preparation System, Illumina, USA).

### 16S data processing and analysis

We will follow the data processing and the analytical pipeline developed by the MiBioGen consortium [71], comprising the following steps: 16S data processing, genotype data processing, and genome-wide association study (GWAS) [71]. For the 16S data processing, we will use the Ribosomal Database Project (RDP) Classifier instead of OTU picking, since it leads to more consistent results and for the genotype imputation we will use the freely available Michigan Imputation Server [72]. For the GWAS analysis we will follow the uniform analytical pipeline developed by the consortium.

### Statistical analyses and power calculation

The association between microbiota composition and NAFLD will be assessed using the Fisher’s exact test for proportions. We built power scenarios using the “power two-proportions” command with the “test(fisher)” option implemented in Stata version 14. The impact of predictor variables on the presence of NAFLD or NAFLD-related symptoms and markers will be determined using univariable and multivariable logistic regression models. The role of T2D in the microbiome-NAFLD relationship will be investigated by means of interaction analyses. To assess the relationship between NAFLD severity and microbiota we will fit linear regression models. All models will be further controlled for participants’ relatedness to avoid biased estimates due to population structure. Finally, we will analyze the difference between microbiota measured from saliva and from stools in participants with NAFLD and in those with T2D.

### Ethical considerations

The CHRIS-NAFLD study protocol was approved by the Ethical Committee of the Healthcare System of the Autonomous Province of Bolzano (Südtiroler Sanitätsbetrieb/Azienda Sanitaria dell’Alto Adige), protocol no. 85-2016 (19 Oct 2016). As it is nested within the CHRIS study, the CHRIS-NAFLD study follows the CHRIS’s ethics protocols with regard to the collection, use and access of data and biosamples, which are stored for a long-term (30 years) after the end of recruitment. Participants were recruited based on a previous consent that allowed re-call. The CHRIS study uses online dynamic consent procedures for empowering the autonomy and compliance of study participants. The dynamic consent options for re-contact, allowed us to re-invite the participants for the CHRIS-NAFLD study, collecting additional information and re-consent. Prior to participation, participants were informed about the objectives and extra procedures of this additional study, for which they provided written informed consent.

## Results

Recruitment took place between October 2016 and February 2017. Out of 454 invited individuals, 356 were recruited (78.4% participation rate). Participation rate was similar in the T2D (173 out of 227 invited participants) and non-T2D (183 out of 227 invited participants) groups. At baseline, the 356 individuals who accepted to participate in the CHRIS-NAFLD study, were on average 67.6 years old (standard deviation, SD = 10.6) and 177 (49.7%) were females. One participant selected in the non-T2D group reported an incident T2D diagnosis at the time of the CHRIS-NAFLD examination and was thus included in the T2D group. We collected saliva and stool samples of 354 and 350 participants, respectively. The DNA extraction and quality control were completed for all samples and they all amplified in PCR. DNA was normalized to ~ 100 ng/µl and stored at − 80 °C until further use.

Characteristics of the 356 participants at the time of participation to the CHRIS-NAFLD study are described in Table 1, following the stratification by T2D status that was used for recruitment. Briefly, 173 (48.6%) and 183 (51.4%) were T2D and non-T2D, respectively. Mean follow-up time was 3.56 years (SD = 0.82) and 3.91 years (0.88) for T2D and non-T2D participants, respectively. As expected, T2D and non-T2D participants had similar age and sex distributions. Of the T2D participants, 8.1% reported to have never consumed alcohol, while 22.5% drink daily (5.5% and 29.5% in the non-T2D group). Most of the participants were never (60.5% and 62.3% for T2D and non-T2D, respectively) or past smokers (32.0% and 32.2%).

**Table 1:** Description of the CHRIS-NAFLD study sample

	Groups	
	T2D^a^ (N = 173)	Non-T2D^a^ (N = 183)
Female—n (%)	84 (48.6)	93 (50.8)
Age (years)—mean (SD)	67.3 (10.4)	67.9 (10.7)
Alcohol consumption (g/day)—median (IQR)	2.0 (0.0, 13.5)	4.9 (1.0, 14.2)
Smoking habits—n (%)		
Never	104 (60.5)	114 (62.3)
Past	55 (32.0)	59 (32.2)
Current	13 (7.6)	10 (5.5)
Self-reported diabetes—n (%)	111 (64.2)	1 (0.6)
Diabetes treatment—n (%)	88 (51.8)	0 (0.0)
Systolic blood pressure—mean (SD)	142.1 (19.6)	137.6 (8.7)
Anti-hypertensive treatment—n (%)	109 (63.4)	60 (32.8)
Lipid-lowering agents—n (%)	77 (45.3)	35 (19.9)
Glycated hemoglobin (%)—median (IRQ)	6.4 (6.0, 6.9)	5.3 (5.2, 5.4)
Fasting glucose (mg/dl)—median (IRQ)	122.5 (107.0, 142.0)	90.0 (85.0, 96.0)
Total cholesterol (mg/dl)—mean (SD)	199.5 (43.7)	215.7 (45.9)
HDL (mg/dl)—mean (SD)	53.2 (12.1)	58.6 (13.6)
LDL (mg/dl)—mean (SD)	125.9 (40.2)	135.9 (40.8)
Triglycerides (mg/dl)—median (IRQ)	111.5 (87.5, 143.5)	91.0 (71.0, 122.0)
Proton pump inhibitors—n (%)	19 (11.2)	17 (9.7)
Statins—n (%)	75 (44.1)	35 (19.9)
Body-mass-index (kg/m^2^)—median (IQR)	30.0 (26.35, 32.7)	25.8 (23.65, 29.0)
Waist circumference (cm)—mean (SD)	100.2 (13.9)	89.5 (12.1)
Body Fat (%)—mean (SD)	35.0 (9.6)	31.1 (10.1)
Visceral Fat (%)—mean (SD)	13.2 (4.8)	10.5 (4.1)
Metabolic syndrome—n (%)	135 (78.0)	45 (24.6)
Hepatic steatosis—n (%)		
Grade 1	56 (32.4)	115 (62.8)
Grade 2	74 (42.8)	60 (32.8)
Grade 3	43 (24.9)	8 (4.4)
Controlled attenuation parameter (dB/m)—median (IQR)	263 (223, 315)	234 (186, 266)
Liver stiffness (kPa)—median (IQR)	4.8 (3.7, 5.9)	3.9 (3.3, 5.1)
Visceral adiposity index—median (IQR)	1.46 (1.09, 2.16)	1.07 (0.77, 1.64)
NAFLD liver fat score—median (IQR)	0.20 (− 0.70, 1.13)	− 1.90 (–2.25, − 1.24)
Hepatic steatosis score—median (IQR)	41.2 (36.0, 46.0)	34.0 (31.6, 38.1)
Fatty liver index—mean (SD)	41.1 (29.6)	55.2 (28.6)
NAFLD fibrosis score—mean (SD)	− 0.55 (1.30)	− 1.30 (1.17)
Fibrosis (Fib)-4—median (IQR)	1.22 (0.93, 1.71)	1.43 (1.07, 1.80)
NAFLD classification—n (%)	116 (63.7)	66 (36.3)

^a^At CHRIS study baseline

Among T2D individuals, 111 (64.2%) reported a diagnosis of diabetes, 88 (51.8%) were on diabetic treatment at the time of the visit, 42.4% had HbA1c values in the pre-diabetic range (HbA1c 6.0 to 6.4%), while 44.2% had HbA1c values in the diabetic range (HbA1c ≥ 6.5%). Fifty percent of the T2D individuals and 18.3% of the non-T2D individuals were obese (BMI ≥ 30). Hypertension was observed at the time of the visit in 55.9% of the T2D individuals and 44.2% of the non-T2D individuals. Use of proton-pump inhibitors (PPIs), which were shown to potentially influence gut microbial composition [73, 74], had similar distribution in T2D and non-T2D participants, while statins were more common in T2D individuals (Table 1).

**Fig. 1:**
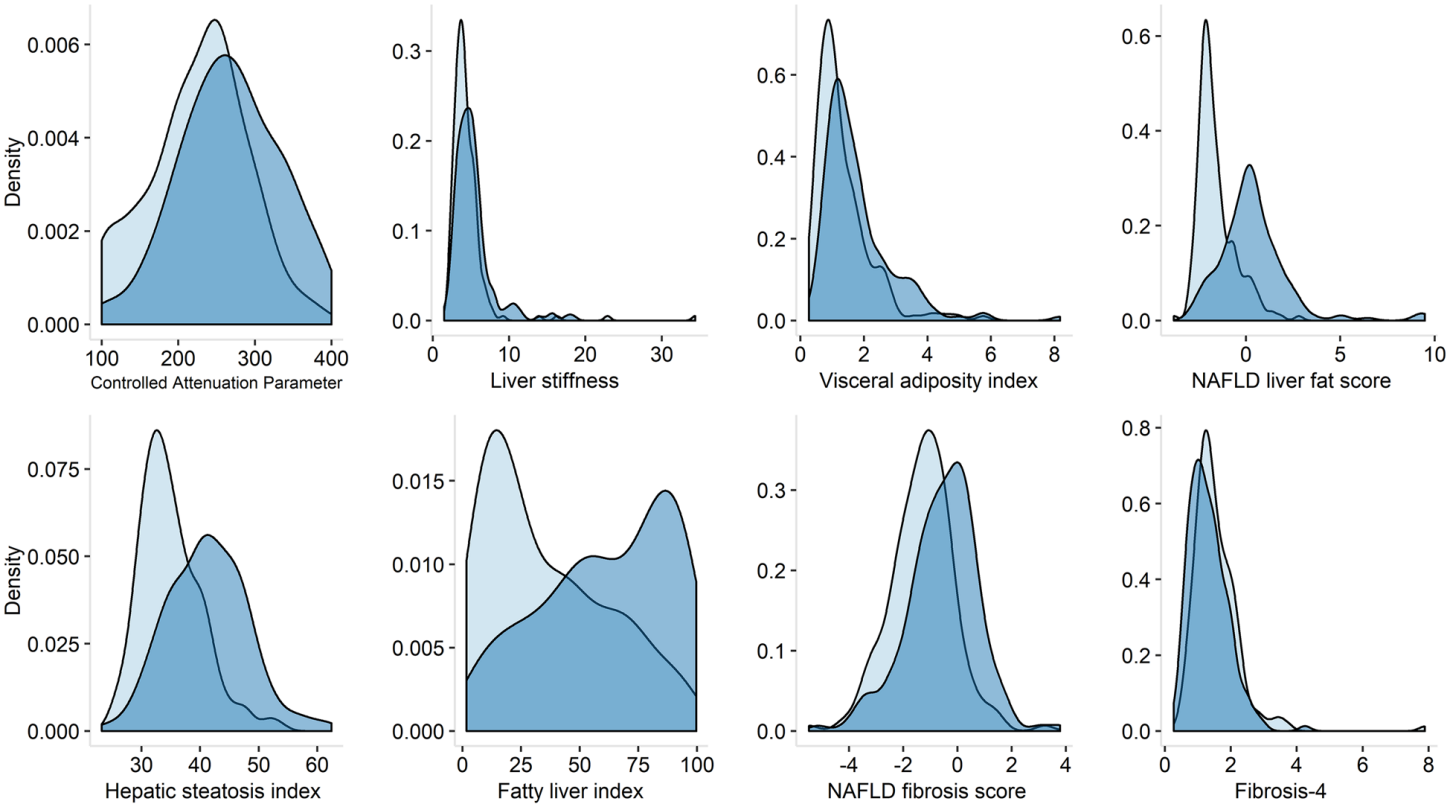
Distribution of NAFLD scores in T2D (dark blue) vs non-T2D (light blue)

Hepatic steatosis was diagnosed in 43 of the 173 T2D individuals (24.9%) and 8 of the 183 non-T2D ones (4.4%). Furthermore, T2D participants showed higher values of TE than non-T2D participants (median = 4.8 (IQR = 3.7, 5.9) kPa vs. 3.9 (3.3, 5.1) kPa) (Fig. 1). These findings agreed with non-invasive scoring systems, like NAFLD fibrosis score, suggesting increased liver fibrosis in T2D individuals (Table 1).

**Fig. 2:**
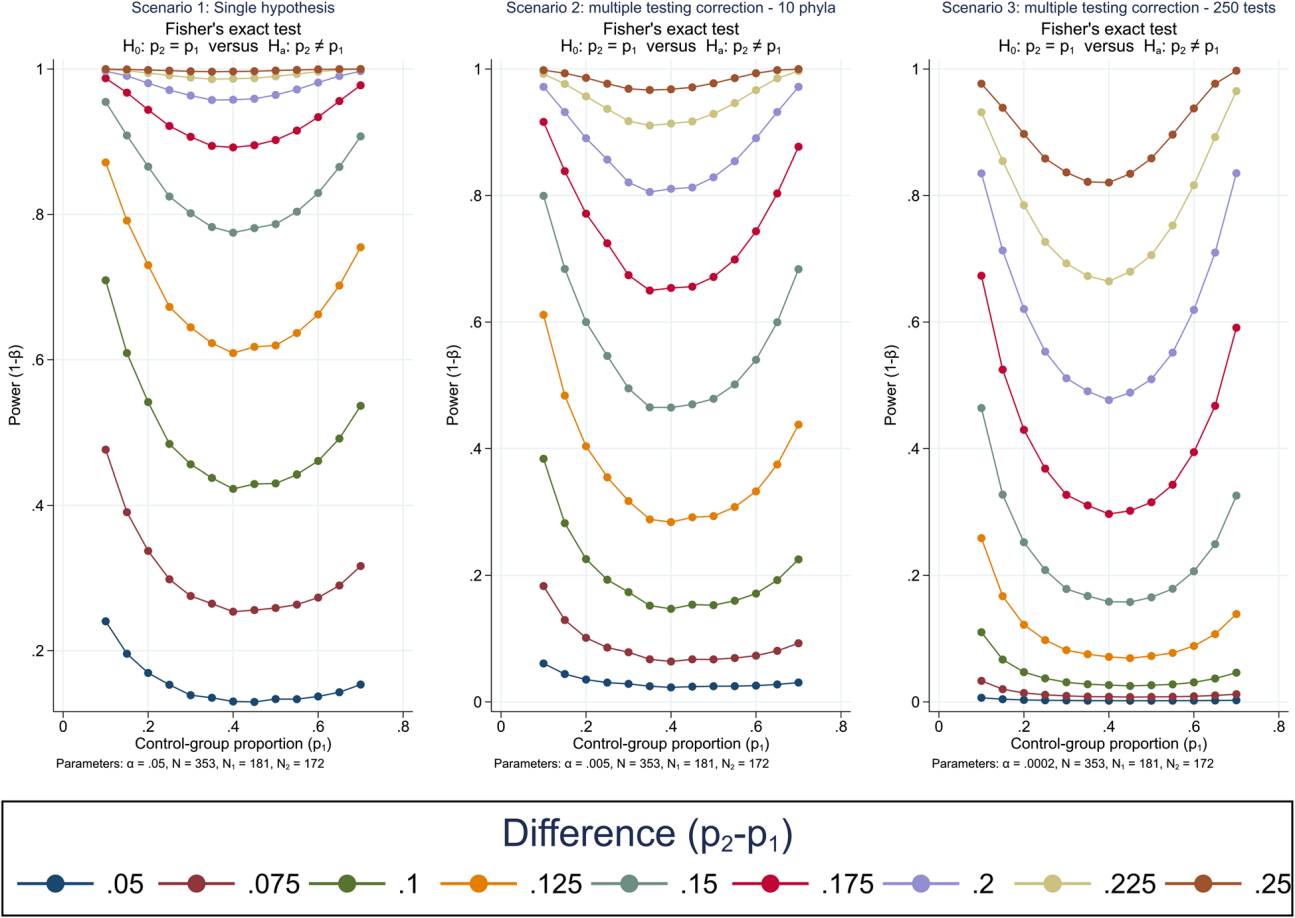
Power to detect given differences between bacterial abundance in 172 NAFLD affected versus 181 non-affected individuals under three different scenarios: a single hypothesis (significance level, α = 0.05), correction for multiple testing of 10 phyla (Bonferroni-corrected α = 0.005), and correction for 250 independent tests (α = 0.0002). p_1_ = proportion of bacteria in non-NAFLD individuals; p_2_ = proportion of bacteria in NAFLD individuals

According to the criteria for NAFLD calculation, we identified 172 as affected with NAFLD and 181 healthy individuals among the two groups of T2D and not-T2D participants. Power calculations are shown in Fig. 2. Assuming we wish to compare the relative bacterial abundance in participants affected with NAFLD versus healthy ones and analyzing data using a Fisher exact test, we anticipate a > 80% power to detect a difference in the proportion of relative abundance of single bacterial strains in the two groups higher than 15% (Fig. 2, scenario 1). Despite previous studies that did not identify more than 6 phyla, we predict finding 10 phyla (Fig. 2, scenario 2); in this scenario, the study will have 80% power to detect differences in the proportions equal or higher than 20%. In Fig. 2, scenario 3, we consider 250 bacterial subgroups, calculated assuming to find 10 phyla, 5 families per phyla and 5 genera per family. Under this scenario the study would have 80% power to detect differences in proportions higher than 22.5% in most cases. However, in terms of multiple comparisons, this should be considered as a pessimistic scenario as none of previous studies was able to identify phyla, families and genera at such a high level of detail.

## Discussion

CHRIS-NAFLD is a population-based study aimed to assess the relationship between gut and oral microbiota and NAFLD in individuals with and without T2D. Stratification by diabetes will enable assessment of whether the NAFLD-microbiota relationship is modified or moderated by, or is independent of, the T2D status. In addition to the cross-sectional nature of the investigation, the study will help to evaluate whether NAFLD assessed at the time of the CHRIS-NAFLD participation was associated with clinical or biochemical information collected at the time of the CHRIS baseline participation. Finally, the study will provide important information on the relationship between microbiota measured at different sites of the human body, specifically from samples of saliva and stool.

Currently, extensive efforts are being focused on the analyses of microbiota in relation to several diseases. In recent years, the importance of gut microbiota in NAFLD was demonstrated by several independent groups. NAFLD patients exhibit distinct changes in their intestinal flora, which impact the host metabolism [75]. Patients show a higher abundance of bacterial strains which supply the host with nutrient source out of indigestible products, such as complex carbohydrates [21, 76]. However, studies conducted in the general population on the association between microbiota and NAFLD are few and with a smaller number of participants compared to ours [32, 77–79].

It is likely that the interaction of genetic and environmental factors with metabolic alterations accelerate NAFLD progression in T2D patients [80]. NAFLD and T2D commonly co-exist and several studies have demonstrated that NAFLD might be found in up to 70% of patients with T2D [6, 81]. From this perspective, our study will allow the question as to whether the NAFLD-microbiota relationship is affected by the diabetic status to be answered.

In a landmark work, Qin and colleagues defined a distinct microbial composition in late stage liver disease. In liver cirrhosis patients, the specific beneficial bacterial strains, like *Faecalibacterium prausnitzii,* were diminished compared to healthy individuals. Additional analyses of buccal flora showed that, in the patients with liver cirrhosis, harmful bacterial strains are transferred from the oral cavity to the intestine, possibly contributing to the development of cirrhosis [43]. In terms of “oralisation” of the intestinal microbiota, the widespread use of PPIs, which reduce the barrier function of gastric acid [74], received great attention in the recent years. Several studies analyzed the impact of PPIs on liver diseases such as hepatic encephalopathy [82] and alcoholic liver disease [83]. Therefore, there is an urgent need to understand the pathophysiological mechanisms leading to NAFLD and to gain more insight into the role of intestinal and oral microbiota in NAFLD.

In the CHRIS-NAFLD study, we collected both saliva and faeces for microbiota analyses. The oral and gastrointestinal microbiome represents the bulk of the overall human microbial load. The correlation of oral microbiota and gut microbiota in NAFLD patients has not been evaluated yet. This will give novel insights into the composition of the microbiota in individuals with and without T2D, possibly identifying microbial transfer in NAFLD patients. Moreover, since saliva is easier to collect compared to stool, if we will observe a similar composition of salivary and stool microbiota, this would increase the compliance of individual participation in such microbiota studies.

In a preliminary descriptive analysis we observed a higher level of liver stiffness in T2D participants, a finding also reported in other cohorts [84]. We also observed a higher prevalence of NAFLD-affected individuals in the T2D groups, as reported by other studies [6, 7].

Strengths of this study include comprehensive assessment of NAFLD, T2D and microbiota in individuals from the general population submitted to a comprehensive evaluation of their hepatic health via ultrasound and elastography examination. In addition to the described data, a wealth of additional genetic, molecular, clinical, environmental data and biological biobanked samples collected in the framework of the CHRIS study are available [44, 45, 85]. Our study has also potential limitations. Even though liver biopsy represents the gold standard for the diagnosis of fibrosis, we used TE for this assessment since the use of an invasive procedure, such as biopsy, would not be ethically justifiable in a population-based observational study. Furthermore, TE is considered a valid noninvasive alternative for this assessment, as reported previously [86]. The ultrasound based method used for detection of steatosis has 85% sensitivity and 94% specificity for identifying a degree of ≥ 20–30% steatosis [87]. Finally, we classified participants that had fasting HbA1c levels of ≥ 6.5% as having T2D since the recent International Expert Committee’s statements recommended using these HbA1c levels as diagnostic criteria for diabetes [46], but there are also some potential factors that can lead to altered HbA1c levels such as chronic salicylate intake in some individuals [88]. Furthermore, the daily medication of participants was also evaluated in our study. PPIs were equally distributed in T2D and non-T2D individuals. In a large meta-analysis, PPIs were shown to be associated with increased enteric infection with *Clostridium difficile* [89]. Statins, which were also proven to influence gut microbiota in mice [90, 91], were more commonly reported by T2D individuals. This difference could be explained by higher numbers of dyslipidemia in T2D participants.

In summary, by combining comprehensive bio-sampling with clinical characterization including detailed information on drug history of a large group of individuals with or without T2D and related NAFLD, the CHRIS-NAFLD study will help elucidate important questions on the relationship between microbiota and presence of NAFLD in patients with and without T2D.

## Data Availability

The datasets generated and/or analyzed during the current study are available from the corresponding author on reasonable request.

## Abbreviations

ALT: alanine transaminase
AST: aspartate transaminase
ATC: anatomical therapeutic chemical
BMI: body mass index
BP: blood pressure
BSS: Bristol stool scale
CHRIS: Cooperative Health Research In South Tyrol
DM: diabetes mellitus
FIB-4: fibrosis-4
FFQ: Food Frequency Questionnaire
FLI: fatty liver index
FPG: fasting plasma glucose
GGT: gamma-glutamyl transferase
GWAS: genome-wide association study
HbA1c: glycated hemoglobin
HDL: high-density lipoproteins
HIS: hepatic steatosis score
HOMA-IR: homeostatic model assessment-insulin resistance
IFG: impaired fasting glucose
IQR: interquartile range
LFS: NAFLD liver fat score
MetS: metabolic syndrome
NAFLD: non-alcoholic fatty liver disease
NASH: non-alcoholic steatohepatitis
NFS: NAFLD fibrosis score
OHQ: Oral Health Questionnaire
PLT: platelets
PPIs: proton-pump inhibitors
RDP: ribosomal database project
SCFAs: short chain fatty acids
SD: standard deviation
T2D: type 2 diabetes
TE: transient elastography
TG: triglyceride
VAI: visceral adiposity index
WC: waist circumference
